# Supplementary material for: BrainSpace: a toolbox for the analysis of macroscale gradients in neuroimaging and connectomics datasets
Source: Commun Biol. 2020 Mar 5;3:103. doi: 10.1038/s42003-020-0794-7 (PMC7058611; doi:10.1038/s42003-020-0794-7)
Supplement: Supplementary file 1 — Supplementary Information [file 42003_2020_794_MOESM1_ESM.pdf]

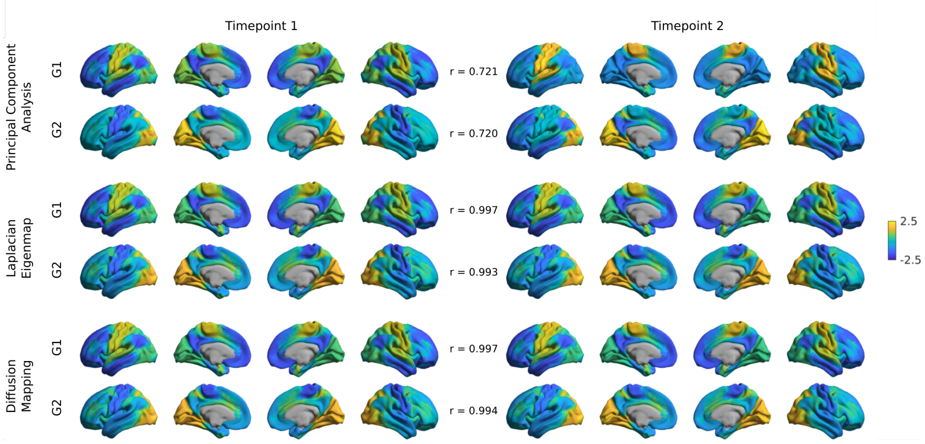


Supplementary Figure 1: Test-retest stability of gradients. Resting-state scans taken on two different days of the same subjects were used to construct separate (unaligned) gradients. Pearson correlation between these are shown.

Supplemental Sample Code 1: Python version for Matlab Sample Code 1. A minimal example for plotting the first gradient of an input data matrix on the cortical surface.

```
from brainspace.gradient import GradientMaps
from brainspace.plotting import plot_hemispheres
from brainspace.mesh import mesh_io as mio

# Create a GradientMaps object
G = GradientMaps(kernel='cosine', approach='dm')

# Apply GradientMaps to the data
G.fit(data_matrix)

# Load surfaces
left_surface = mio.read_surface('left_surface_file.obj')
right_surface = mio.read_surface('right_surface_file.obj')

# Plot first gradient on the cortical surface
plot_hemispheres(left_surface, right_surface, G.gradients_[:,0])
```

Supplemental Sample Code 2: Python version for Matlab Sample Code 2. A minimal example for creating and plotting gradients from different modalities, with different alignments.

```
from brainspace.gradient import GradientMaps
from brainspace.plotting import plot_hemispheres
from brainspace.mesh import mesh_io as mio
```

```python
# Create two GradientMaps objects with different alignments
Gp = GradientMaps(kernel='cosine', approach='dm',
                  alignment='procrustes')
Gj = GradientMaps(kernel='cosine', approach='dm',
                  alignment='joint')

# Apply GradientMaps to the data
Gp.fit([mpc, fc])
Gj.fit([mpc, fc])

# Load surfaces
left_surface = mio.read_surface('left_surface_file.obj')
right_surface = mio.read_surface('right_surface_file.obj')

# Plot first MPC gradient of Procrustes alignment
plot_hemispheres(left_surface, right_surface, Gp.aligned_[0][:,0])

# Plot first FC gradient of joint embedding
plot_hemispheres(left_surface, right_surface, Gj.aligned_[1][:,0])
```

Supplemental Sample Code 3: Python version for Matlab Sample Code 3. A minimal example for aligning the gradients of two individuals to a template gradient.

```python
from brainspace.gradient import GradientMaps
from brainspace.plotting import plot_hemispheres
from brainspace.mesh import mesh_io as mio

# Create a GradientMaps object for the template
Gt = GradientMaps(kernel='cosine', approach='dm')

# Apply to template data
Gt.fit(template_data)

# Create a GradientMaps object for the individuals
Gs = GradientMaps(approach='dm', kernel='cosine',
                  alignment='procrustes')

# Compute gradients for all subjects and align to template
Gs.fit([subject1_data, subject2_data], reference=Gt.gradients_)

# Load surfaces
left_surface = mio.read_surface('left_surface_file.obj')
right_surface = mio.read_surface('right_surface_file.obj')

# Plot the first aligned gradient for subject 2
plot_hemispheres(left_surface, right_surface, Gs.aligned_[1][:, 0])
```

Supplemental Sample Code 4: Python version for Matlab Sample Code 4. A minimal Python example for building null models based on spin tests.

```python
from brainspace.null_models import SpinPermutations
from brainspace.mesh import mesh_io as mio

# Load spheres
left_sphere = mio.read_surface('left_sphere_file.obj')
right_sphere = mio.read_surface('right_sphere_file.obj')

# Generate 1000 spin permutations
n_perm = 1000
sp = SpinPermutations(n_rep=n_perm)
sp.fit(left_sphere, points_rh=right_sphere)
features_spin = sp.randomize(left_features, x_rh=right_features)
```
