# Supplementary material for: BrainSpace: a toolbox for the analysis of macroscale gradients in neuroimaging and connectomics datasets
Source: Commun Biol. 2020 Mar 5;3:103. doi: 10.1038/s42003-020-0794-7 (PMC7058611; doi:10.1038/s42003-020-0794-7)
Supplement: Supplementary file 2 — Reporting Summary [file 42003_2020_794_MOESM2_ESM.pdf]

nature research

Corresponding author(s): Boris C. Bernhardt

Last updated by author(s): 16/1/20

Reporting Summary

Nature Research wishes to improve the reproducibility of the work that we publish. This form provides structure for consistency and transparency in reporting. For further information on Nature Research policies, see Authors & Referees and the Editorial Policy Checklist.

Statistics

For all statistical analyses, confirm that the following items are present in the figure legend, table legend, main text, or Methods section.

n/a	Confirmed	
☐	☒	The exact sample size (n) for each experimental group/condition, given as a discrete number and unit of measurement
☒	☐	A statement on whether measurements were taken from distinct samples or whether the same sample was measured repeatedly
☐	☒	The statistical test(s) used AND whether they are one- or two-sided<br>*Only common tests should be described solely by name; describe more complex techniques in the Methods section.*
☒	☐	A description of all covariates tested
☐	☒	A description of any assumptions or corrections, such as tests of normality and adjustment for multiple comparisons
☐	☒	A full description of the statistical parameters including central tendency (e.g. means) or other basic estimates (e.g. regression coefficient) AND variation (e.g. standard deviation) or associated estimates of uncertainty (e.g. confidence intervals)
☐	☒	For null hypothesis testing, the test statistic (e.g. F, t, r) with confidence intervals, effect sizes, degrees of freedom and P value noted<br>*Give P values as exact values whenever suitable.*
☒	☐	For Bayesian analysis, information on the choice of priors and Markov chain Monte Carlo settings
☒	☐	For hierarchical and complex designs, identification of the appropriate level for tests and full reporting of outcomes
☒	☐	Estimates of effect sizes (e.g. Cohen's d, Pearson's r), indicating how they were calculated

Our web collection on statistics for biologists contains articles on many of the points above.

Software and code

Policy information about availability of computer code

Data collection	This manuscript uses public data from the Human Connectome Project dataset available at https://db.humanconnectome.org. Availability of software used in data collection is responsibility of the original submitters to the repository.
Data analysis	Data analysis as shown in the manuscript was performed in MATLAB R2018b. The analysis package used as well as a Python3 equivalent is available at https://github.com/MICA-MNI/BrainSpace under an open-source license (BSD-3-clause).

For manuscripts utilizing custom algorithms or software that are central to the research but not yet described in published literature, software must be made available to editors/reviewers. We strongly encourage code deposition in a community repository (e.g. GitHub). See the Nature Research guidelines for submitting code & software for further information.

Data

Policy information about availability of data

All manuscripts must include a data availability statement. This statement should provide the following information, where applicable:

- Accession codes, unique identifiers, or web links for publicly available datasets
- A list of figures that have associated raw data
- A description of any restrictions on data availability

All data are freely provided by the Human Connectome Project (Van Essenet al., 2013) and available from connectomeDB (Hodge et al., 2016) (https://db.humanconnectome.org/).

Field-specific reporting

Please select the one below that is the best fit for your research. If you are not sure, read the appropriate sections before making your selection.

☒ Life sciences ☐ Behavioural & social sciences ☐ Ecological, evolutionary & environmental sciences

For a reference copy of the document with all sections, see nature.com/documents/nr-reporting-summary-flat.pdf

Life sciences study design

All studies must disclose on these points even when the disclosure is negative.

Sample size	The same sample was used as in a prior study (Vos de Wael et al., 2018, PNAS). In short, subjects with complete T1w, T2w, and resting-state fMRI were selected. These subjects were split into two groups of unrelated subjects. One of these groups (N=217) was used for this study. For the microstructural profile covariance analyses we selected subjects both in this dataset as well as the dataset described by (Paquola et al, PLOS Biology; N=70).
Data exclusions	No exclusions beyond the critera mentioned above
Replication	Sample functional connectivity matrices are provided alongside with the proposed toolbox for the reproduction of cortical gradients.
Randomization	N/A; only a single group was used in this study.
Blinding	N/A; only a single group was used i.e. no blinding was possible.

Reporting for specific materials, systems and methods

We require information from authors about some types of materials, experimental systems and methods used in many studies. Here, indicate whether each material, system or method listed is relevant to your study. If you are not sure if a list item applies to your research, read the appropriate section before selecting a response.

Materials & experimental systems

n/a	Involved in the study	
☒	☐	Antibodies
☒	☐	Eukaryotic cell lines
☒	☐	Palaeontology
☒	☐	Animals and other organisms
☐	☒	Human research participants
☒	☐	Clinical data

Methods

n/a	Involved in the study	
☒	☐	ChIP-seq
☒	☐	Flow cytometry
☐	☒	MRI-based neuroimaging

Human research participants

Policy information about studies involving human research participants

Population characteristics	Population characteristics were not used as covariates.
Recruitment	Recruitment was performed by the HCP consortium. The primary recruitment pool was from families with twins, but it was supplemented by additional recruitment such that the participants reflected the ethnic and racial composition of the U.S population.
Ethics oversight	Ethics approval for the acquisition and uploading of the data was requested by and granted to the HCP consortium.

Note that full information on the approval of the study protocol must also be provided in the manuscript.

Magnetic resonance imaging

Experimental design

Design type	Structural Scans (T1w and T2w) as well as resting-state fMRI
Design specifications	The method presented herein requires a 2D input matrix of any kind (although we present functional connectivity throughout the manuscript) and cortical surfaces for visualization.
Behavioral performance measures	N/A

Acquisition

Imaging type(s)	T1w, T2w, Resting-State fMRI
Field strength	3T
Sequence & imaging parameters	T1w images were acquired using a 3D-MPRAGE sequence (0.7mm isotropic voxels, matrix=320́ 320, 256 sagittal slices; TR=2400ms, TE=2.14ms, TI=1000ms, flip angle=8°; iPAT=2). T2w images were acquired with identical geometry (TR=3200ms, TE=565ms, variable flip angle; iPAT=2). rs-fMRI scans were acquired using multi-band accelerated 2D-BOLD echo-planar imaging (2mm isotropic voxels, matrix=104́ 90, 72 sagittal slices; TR=720ms, TE=33ms, flip angle=52°; mb factor=8; 1200 volumes/scan).
Area of acquisition	Whole brain

Diffusion MRI ☐ Used ☒ Not used

Preprocessing

Preprocessing software	Preprocessing was performed by the HCP consortium as described in the HCP S900 Manual. Packages used were FSL 5.0.6, Freesurfer 5.3.0-HCP, Connectome Workbench V1.1.1
Normalization	Non-linear normalization to the ICBM152 template was performed.
Normalization template	Subjects were normalized to the ICBM152 template.
Noise and artifact removal	Timeseries were corrected for gradient nonlinearity. Head motion was corrected using rigid body transformation. The R-L/L-R blipped scan pairs were used to correct geometric distortions. A high-pass filter (>2000s FWHM) corrected the time series for scanner drifts, and additional noise was removed using the ICA-FIX procedure
Volume censoring	No volume censoring was performed.

Statistical modeling & inference

Model type and settings	Statistical models are described in detail in the manuscript. In short we use spin test which rotates the data on the cortical sphere and propose Moran spectral randomization which generates new data with comparable spatial auto-correlation.
Effect(s) tested	Correspondence between functional connectivity derived gradients and other markers, specifically: microstructural profile covariance, T1w/T2w intensity, and cortical thickness.

Specify type of analysis: ☒ Whole brain ☐ ROI-based ☐ Both

Statistic type for inference (See Eklund et al. 2016)	N/A; the manuscript proposes the use of two null models for inference on gradient data.
Correction	N/A; statistical tests are shown as examples of the proposed method, rather than to determine significance.

Models & analysis

n/a	Involved in the study
☐	☒ Functional and/or effective connectivity
☒	☐ Graph analysis
☒	☐ Multivariate modeling or predictive analysis

Functional and/or effective connectivity	Functional connectivity was computed with a Pearson correlation.
